# Bcl-2-like protein-10 increases aggressive features of melanoma cells

**DOI:** 10.37349/etat.2022.00068

**Published:** 2022-01-30

**Authors:** Donatella Del Bufalo, Marta Di Martile, Elisabetta Valentini, Isabella Manni, Ilenia Masi, Antonella D’Amore, Antonio Filippini, Carmine Nicoletti, Marco Zaccarini, Carlo Cota, Maria Victoria Castro, María Josefina Quezada, Laura Rosanò, Pablo Lopez-Bergami, Simona D’Aguanno

**Affiliations:** 1 Preclinical Models and New Therapeutic Agents Unit, IRCCS Regina Elena National Cancer Institute, 00144 Rome, Italy; 2 SAFU Unit, IRCCS Regina Elena National Cancer Institute, 00144 Rome, Italy; 3 Institute of Molecular Biology and Pathology, National Research Council, 00161 Rome, Italy; 4 Unit of Histology and Medical Embryology, Department of Anatomy, Histology, Forensic Medicine and Orthopaedics, Sapienza University, 00161 Rome, Italy; 5 Genetic Research, Dermatological Molecular Biology and Dermatopathology Unit, IRCCS San Gallicano Dermatological Institute, 00144 Rome, Italy; 6 Centro de Estudios Biomédicos, Básicos, Aplicados y Desarrollo, Universidad Maimónides, Buenos Aires C1405BCK, Argentina; 7 Consejo Nacional de Investigaciones Científicas y Técnicas, Buenos Aires C1405BCK, Argentina; Université de Lyon, France

**Keywords:** B-cell lymphoma-2-like protein-10, melanoma, invasion, migration, vasculogenic mimicry

## Abstract

**Aim:**

B-cell lymphoma-2 (Bcl-2)-like protein-10 (Bcl2L10) is the less studied member of Bcl-2 family proteins, with the controversial role in different cancer histotypes. Very recently, Bcl2L10 expression in melanoma tumor specimens and its role in melanoma response to therapy have been demonstrated. Here, the involvement of Bcl2L10 on the *in vitro* and *in vivo* properties associated with melanoma aggressive features has been investigated.

**Methods:**

Endogenous Bcl2L10 protein expression was detected by western blotting analysis in a panel of patient-derived and commercially available human melanoma cells. *In vitro* assays to evaluate clonogenicity, cell proliferation, cell migration, cell invasion, and *in vitro* capillary-like structure formation [vasculogenic mimicry (VM)] have been performed by using human melanoma cells stably overexpressing Bcl2L10 or transiently transfected for loss/gain function of Bcl2L10, grown under two- or three-dimensional (3D) conditions Xenograft melanoma model was employed to evaluate *in vivo* tumor growth and angiogenesis.

**Results:**

Results demonstrated that Bcl2L10 acts as an inducer of *in vitro* cell migration, invasion, and VM, while *in vitro* cell proliferation, *in vivo* tumor growth, as well as colony formation properties were not affected. Dissecting different signaling pathways, it was found that Bcl2L10 positively affects the phosphorylation of extracellular-signal-regulated kinase (ERK) and the expression of markers of cell invasion, such as urokinase plasminogen activator receptor (uPAR) and matrix metalloproteinases (MMPs). Of note, Bcl2L10-dependent *in vitro* migration, invasion, and VM are linked to uPAR. Bcl2L10 also negatively regulates the intracellular calcium level. Finally, reduced invasion capability in 3D spheroid invasion assay of melanoma cells transiently overexpressing Bcl2L10 was observed after treatment with inhibitors of MMPs and uPAR.

**Conclusions:**

Overall, data reported in this paper provide evidence supporting a positive role of Bcl2L10 in melanoma aggressive features.

## Introduction

Cutaneous melanoma is one of the most aggressive forms of skin cancer, characterized by high mortality rate, metastases, and resistance to conventional therapies [1]. Although the most frequent driver mutations have been discovered, pathogenesis and mechanisms of melanoma progression have not been completely elucidated so far. Several cellular pathways were described to be activated during melanoma progression, such as rat sarcoma (RAS)/v-raf murine sarcoma viral oncogene homolog B1 (BRAF)/mitogen-activated protein kinases (MAPK) and phosphatidylinositol 3-kinase (PI3K)/AKT, have been reported to promote or downregulate different B-cell lymphoma-2 (Bcl-2) family molecules. Bcl-2 family members are mainly involved in the control of intrinsic apoptotic pathways by regulating mitochondrial integrity and caspases activation. They are expressed in normal melanocytes and are frequently deregulated in melanoma [2]. Previously studies found that both Bcl-2 and Bcl-xL, antiapoptotic members of the Bcl-2 family, were associated with melanoma progression, resistance to apoptosis, and poor prognosis by acting on processes, such as cell proliferation, migration, invasion, and activating crosstalk with the tumor microenvironment [2–4].

Bcl-2-like protein-10 (Bcl2L10), also known as Bcl-B and NrH, is one of the less studied and latest identified proteins belonging to Bcl-2 family [5–7]. It is widely expressed in human tissues and normal plasma cells [8]. Bcl2L10 is also evidenced in tumors from different origins, including breast, prostate, gastric and colorectal adenocarcinomas, as well as non-small cell lung cancer and papillary thyroid carcinoma [8, 9]. *Bcl2L10* expression in hematological malignancies, such as multiple myeloma and diffuse large B-cell lymphoma, was also found [8, 10]. Very recently, high Bcl2L10 expression both in melanoma cell lines and specimens from melanoma patients has been reported [11]. An association of Bcl2L10 expression with poor prognosis in breast, prostate, colorectal, and small cell lung cancer has been also evidenced [8, 12]. On the contrary, Bcl2L10 correlated with a better outcome in gastric cancer where it works as a tumor suppressor; moreover, hypermethylation of the gene promoter has been found to play an important role in silencing its expression [8, 13, 14].

Bcl2L10 shows both pro- and anti-apoptotic roles, which are related to cell or tissue context [5, 6, 15–17]. In some cases, Bcl2L10 was even reported not to affect apoptosis of cancer cells, thus indicating that in several models it may not be a regulator of apoptosis [18]. A different effect on apoptosis has been proposed to be related to the endogenous expression level of Bcl2L10 protein [19]. Inhibition of calcium release from the endoplasmic reticulum by Bcl2L10 has been reported as the cause of its anti-apoptotic activity [12]. Very recently, the ability of Bcl2L10 to protect melanoma cells from the cytotoxic effect of different drugs has been demonstrated [11]. The involvement of Bcl2L10 in suppressing autophagic cell death caused by several stimuli, in a beclin-1-dependent manner, has also been reported in gastric and cervical cancer [17, 20, 21].

Polyubiquitination and proteasomal turnover, as well as gene amplification, have been identified as relevant mechanisms dictating Bcl2L10 expression [22–24]. Ubiquitin-dependent proteasomal degradation has been found to affect Bcl2L10 function as regulator of apoptosis, while increased expression of *Bcl2L10* by gene amplification has been linked to acquired drug resistance in cancer cells [23, 24]. Bcl2L10 also represents a predictive factor for resistance to azacitidine in myelodysplastic syndromes and acute myeloid leukemia patients [25], and for response to neoadjuvant chemoradiotherapy in locally advanced rectal cancer [9] or to death-inducing agents in breast cancer [12]. The analysis of the prevalence of Leucine21Arginine polymorphism of Bcl2L10 in patients previously treated with radiotherapy and/or chemotherapy evidenced that patients with this polymorphic variant show a decreased risk to develop therapy-related myeloid neoplasms and *de novo* myelodysplastic syndromes [26]. Bcl2L10 has been also reported to participate in tumorigenicity through regulation of invasive/migratory ability of ovarian cancer, as well as angiogenesis and metastatization of hepatocellular carcinoma [9, 13]. Moreover, as evidenced for other pro-survival Bcl-2 family proteins [27], Bcl2L10 cooperates with Myc to induce leukemogenesis [22].

As at present, there is no evidence about the involvement of Bcl2L10 in melanoma progression, the current study aims at investigating whether Bcl2L10 affects properties known to be associated with tumor aggressiveness, such as *in vitro* and *in vivo* tumor growth as well as migration, invasion and capillary-like structure formation [vasculogenic mimicry (VM)].

## Materials and methods

### Cell culture

M14, A375 and WM115 human melanoma cell lines were purchased from American Type Culture Collection (Manassas, VA, USA). SBCL1 human melanoma cell line was provided by Dr. B.C. Giovanella [28]. Patient-derived human melanoma cells ME70, ME10538, ME1007, ME4405 and ME14464 were provided by Dr. A. Anichini [29]. All cell lines were grown in Roswell Park Memorial Institute (RPMI) medium (Euroclone, Milan, Italy) containing 10% (*v*/*v*) fetal bovine serum (FBS), 1% penicillin/streptomycin and 1% *l*-glutamine (Euroclone) in a balanced air humidified incubator with 5% CO_2_ and at 37°C. M14, A375, WM115, ME10538, ME14464 carried BRAF mutation, while ME1007 and ME4405 were BRAF wild type.

M14, A375, SBCL1 and ME4405 cell lines were authenticated using high-throughput single nucleotide polymorphisms (SNP)-based assays. Authentication for the other cell lines is ongoing. Experiments were performed with mycoplasma-free cells.

*Bcl2L10* stably overexpressing clones were obtained transfecting 1.5 × 10^5^ M14 cells with empty vector or *Bcl2L10* vector expressing the full-length human Bcl2L10 protein fused to the N-terminal Myc epitope tag [11]. Jet-PRime reagents (Polyplus Co., Illkirch, France) were used according to the manufacturer’s protocol. Control (M14-C) and *Bcl2L10* overexpressing (M14-B) clones were then selected in the presence of 1,200 μg/mL geneticin. For transient transfection, 1.5 × 10^5^ A375 cells were transfected with empty or *Bcl2L10* expressing vectors, or with pooled oligonucleotide mix against *Bcl2L10* or scramble target sequences [20 nmol/L si-Bcl2L10 or si-Ctrl (Dharmacon RNA Technologies, siGENOME SMARTpool, Lafayette, CO, USA) using INTERFERin® (Polyplus Co.)] according to the manufacturer’s instructions. Control (A375-C), *Bcl2L10* overexpressing (A375-B), *Bcl2L10* interfered (si-Bcl2L10), and scramble interfered (si-Ctrl) cells were obtained. After 48 h of transfection, protein expression was evaluated. For urokinase plasminogen activator receptor (uPAR) silencing, 1.5 × 10^5^ *Bcl2L10* overexpressing M14 cells were transfected with a 20 nmol/L si-uPAR or si-Ctrl (Dharmacon RNA Technologies) using INTERFERin® (Polyplus Co.) according to the manufacturer’s instructions. After 48 h of transfection, protein expression was evaluated.

### Intracellular calcium levels determination

For intracellular calcium levels ([Ca^2+^]_i_) determination, M14-C and M14-B stable clones cultured on 35 mm dishes were incubated in culture medium containing 3.5 μmol/L 2-[6-[bis[2-[(Acetyloxy)methoxy]- 2-oxoethyl]amino]-5-[2-[2-[bis[2-[(acetyloxy)methoxy]-2-oxoethyl]amino]-5-methylphenoxy]ethoxy]- 2-benzofuranyl]-5-oxazolecarboxylic acid (acetyloxy)methyl ester (FURA-2-AM, Invitrogen, Carlsbad, California, USA) for 30 min at 37°C, and then rinsed with Hank’s balanced salt solution (Sigma-Aldrich, St. Louis, Missouri, USA). Dishes were placed into a culture chamber on the support of an inverted fluorescence microscope (Nikon TE2000E, Nikon Instruments, Italy), at 37°C connected to a cooled charge-coupled devices camera (12B cascade, Roper Scientific, Ottobrunn, Germany). Random access monochromator was used to illuminate samples alternately at 340 and 380 nm (Photon Technology International, New Jersey, USA). A 510 nm emission filter was used to detect emission. Cells were stimulated with 100 μm histamine. Metafluor^®^ software (Universal Imaging Corporation, Downington PA, USA) was used to acquire images (1 ratio image per s). At the end of each experiment, calibration was obtained by maximally increasing intracellular Ca^2+^-dependent FURA-2-AM fluorescence with 5 μmol/L ionomycin (ionomycin calcium salt from *Streptomyces conglobatus*, Sigma) followed by recording minimal fluorescence in a Ca^2+^-free medium. [Ca^2+^]_i_ was calculated as previously described [30].

### Western blotting analysis and zymography

Cells were lysed in 10 mmol/L trisaminomethane hydrochloride buffer pH 7.4 with 2% sodium dodecyl sulphate (SDS) and fresh protease inhibitors. Protein concentrations were determined by colorimetric assay after extracts sonication for 20 s (Pierce™ BCA Protein Assay Kit, Thermo Scientific, Waltham, Massachusetts, USA). The following primary antibodies were used to perform western blotting: Bcl2L10 (#3869, Cell Signaling, Danvers, MA, USA), p44/42 [extracellular-signal-regulated kinase (ERK)1/2, #9102, Cell Signaling], phosphorylated p44/42 (ERK1/2, #9106 L, Cell Signaling) and MMP2 (H-76, sc-10736, Santa Cruz Biotechnology, Santa Cruz, CA). β-actin (#A1978, Sigma-Aldrich) and heat shock protein (HSP)72/73 (#HSP01, Calbiochem, San Diego, CA, USA) were used to check equivalent transfer and loading. Chemiluminescent method (Pierce, Rockford, IL) was used to detect immunostained bands. Image Lab™ Software (Bio-Rad, Hercules, CA, USA) and ChemiDoc System instrument (Bio-Rad), were used to acquire images, while ImageJ software was used for densitometric evaluation and normalization with relative controls.

Cultured medium (CM) from M14-B and M14-C cells incubated in serum free medium (SFM) for 24 h was normalized to the number of adherent cells and assayed for gelatinase activity using 7.5% SDS gels containing gelatin (0.1 mg/mL) as previously described [31, 32].

### *In vitro* cell migration, invasion, and VM (capillary-like structure formation) assays

Two-dimensional (2D) cell migration and invasion were performed at 37°C for 8 h using ThinCerts (Greiner Bio-one, Kremsmünster, Austria) containing 8 μm pore polycarbonate membrane as previously reported [4, 29, 33]. A total of 5 × 10^4^ cells (M14-C, M14-B, A375-C, A375-B, A375 si-Ctrl, A375 si-Bcl2L10) were seeded in SFM into the upper chamber of ThinCerts. For invasion assay, the ThinCerts were previously coated with basement membrane extract (BME, Cultrex, 8–12 mg/mL, R&D Systems, Minneapolis, MN, USA) diluted 1:25 with cold SFM, and incubated for 1 h at 37°C. The lower well contained medium with 10% FBS (Hyclone, Thermo Scientific, South Logan, UT) After migration or invasion, a cotton swab was used to remove the cells present in the topside of the membrane. Then, migrating/invading cells were fixed and stained by using differential quick stain kit (Dade Behring, Marburg, Germany). Images were acquired by using a Nikon Eclipse Ts100 microscope and Infinity software and then the migrated/invaded cells were counted. At least five images were acquired for each condition. 2D cell migration and invasion assay were also performed in M14 cells stably overexpressing *Bcl2L10* after pharmalogical inhibition of uPAR, using the M25 peptide (YHHLSLGYMYTLN) at 50 μmol/L, as previously reported [33]. M25 peptide has been designed to impair the interaction between integrin α chain and uPAR, thus inactivating the subsequent signaling pathway [34, 35]. In this last condition, images were acquired by using the light channel of Bio-Rad ZOE fluorescent cell imager (Bio-Rad Laboratories).

Analysis of VM was performed as previously reported by using 24-well plates [4, 29, 33]: 250 μL of BME were dropped onto each well and were allowed to solidify in humidified 5% CO_2_ incubator for 1 h at 37°C. A total of 1 × 10^5^ M14 and 1.5 × 10^5^ A375 cells were seeded in SFM onto the gelled BME and incubated at 37°C for 18 h. Capillary-like structures formation was photographed using Nikon Eclipse Ts100 light microscopy and quantified in 10 sets of images for condition. VM formation was also evaluated in M14 cells stably overexpressing *Bcl2L10* after treatment with M25 peptide at 50 μmol/L for 24 h, and acquiring the images by using the light channel of Bio-Rad ZOE fluorescent cell imager (Bio-Rad Laboratories).

### Three-dimensional spheroid invasion assay

Cultrex 3-D spheroid cell invasion assay (Cultrex #3500-096-K) was employed for three-dimensional (3D) invasion assay, following the manufacturer’s instructions. Spheroids of A375 transfected cells were generated as previously reported by plating 500 cells for 48 h in 3D culture qualified 96-well plate [36]. Then, spheroids were embedded into the Cultrex spheroid invasion matrix. After 1 h at 37 °C, SFM with or without broad-spectrum matrix metalloprotease inhibitor, Ilomastat/GM6001 (Cat# GM6001; Millipore, Burlington, Massachusetts, USA) or uPAR inhibitor, M25 peptide, was added to spheroids wells. Plates were incubated for 7 days, and spheroids were photographed every 24 h through the use of Bio-Rad ZOE fluorescent cell imager (Bio-Rad). ImageJ software (https://imagej.nih.gov/ij/) was used to measure invasion area. Captured images were converted to 8 bits, and the threshold was set to capture the total structure and calculate the area of the spheroids.

### Statistical analysis

Results are expressed as mean ± standard deviation (or standard error of the mean where specified) of at least three independent experiments, unless specified. Differences between groups were analyzed with an unpaired two-tailed student’s *t* test with Welch’s correction and considered statistically significant for *P* < 0.05.

## Results

### *Bcl2L10* is expressed in human melanoma cells and regulates intracellular level of calcium

**Figure 1.**
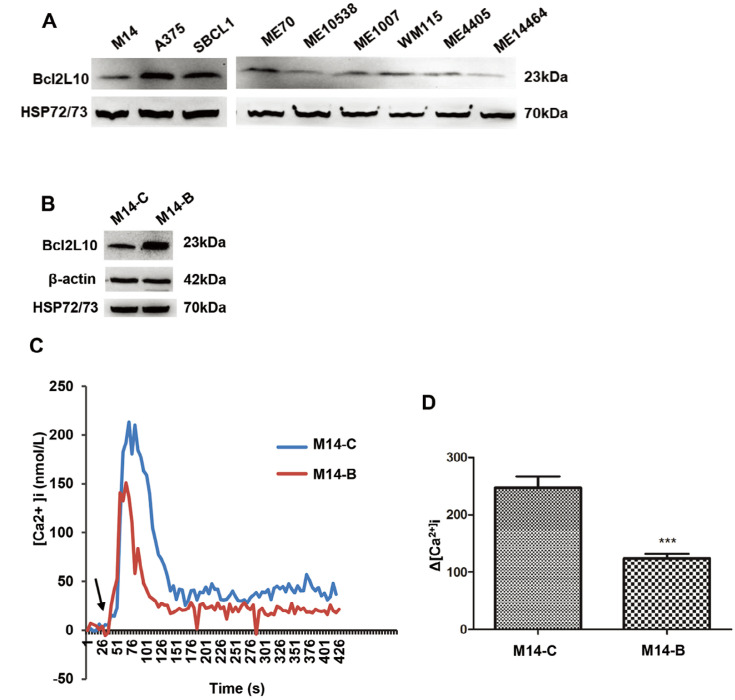
*Bcl2L10* is expressed in human melanoma cells and inhibits intracellular calcium release. (A) Western blot analysis of endogenous Bcl2L10 protein expression in human melanoma cell lines (M14, A375, SBCL1, WM115) and patient-derived melanoma cells (ME70, ME10538, ME1007, ME4405, ME14464). (B) Western blot analysis of Bcl2L10 protein expression in M14 cells stably overexpressing *Bcl2L10* (M14-B) obtained by transfecting M14 parental cells with the plasmid expressing the full length human Bcl2L10 protein fused to the N-terminal Myc epitope tag. Empty vector was used to obtain the control clone (M14-C). Reported images are representative of at least two (A) or three (B) independent experiments with similar results. β-actin and HSP72/73 were used to check equal loading and transfer. (C, D) Live imaging in single FURA-2-AM loaded M14-C and M14-B cells. [Ca^2+^]_i_ were measured after stimulation with histamine. Calcium levels changes are reported as (C) representative traces and (D) as maximum Ca^2+^ concentrations in bar charts. Arrow indicates time of histamine addition. Data were expressed as average ± standard deviation of three independent experiments. *n* = 30–45 cells/experiment. Statistical analysis was performed applying *t* test. *** *P* < 0.001

We first evaluated the endogenous levels of Bcl2L10 protein in a panel of established and primary human melanoma cells [29]. In agreement with our recent findings [11], Bcl2L10 protein was expressed in all cells tested, although with different extent, thus confirming its expression in melanoma cells (Figure 1A). A barely detectable expression of Bcl2L10 protein was also observed in the transformed melanocytes (Figure S1). By interrogating the Human Protein Atlas database (https://www.proteinatlas.org/), Bcl2L10 protein showed cytoplasmic expression in several normal tissues, most abundantly in liver and kidney. It is noteworthy that looking at data reported by the database for single cell type belonging to “skin”, melanocytes are not expressing *Bcl2L10* mRNA (Figure S1). Moreover, no detection has been reported in skin specimens by immunohistochemistry (https://www.proteinatlas.org/), differently to what has been reported for other proteins belonging to Bcl-2 family, such as Bcl-2 and Bcl-xL, which are strongly expressed in normal melanocytes [2].

To assess the functional relevance of Bcl2L10 on *in vitro* melanoma aggression properties, we generated control (M14-C) and *Bcl2L10* overexpressing (M14-B) stably clones from M14 melanoma cells (Figure 1B). As Bcl2L10 has been reported to exert its antiapoptotic activity in breast cancer models through inhibition of calcium release from the endoplasmic reticulum [12], we firstly characterized the contribution of *Bcl2L10* overexpression to calcium release in melanoma model. To achieve this purpose, both control and *Bcl2L10* overexpressing M14 cells were stimulated with histamine, and intracellular calcium was assayed in FURA-2-AM loaded single cells. *Bcl2L10* overexpression inhibited calcium signaling respected to control cells (Figure 1C, 1D).

### Bcl2L10 promotes *in vitro* melanoma cell migration, invasion, and VM

**Figure 2.**
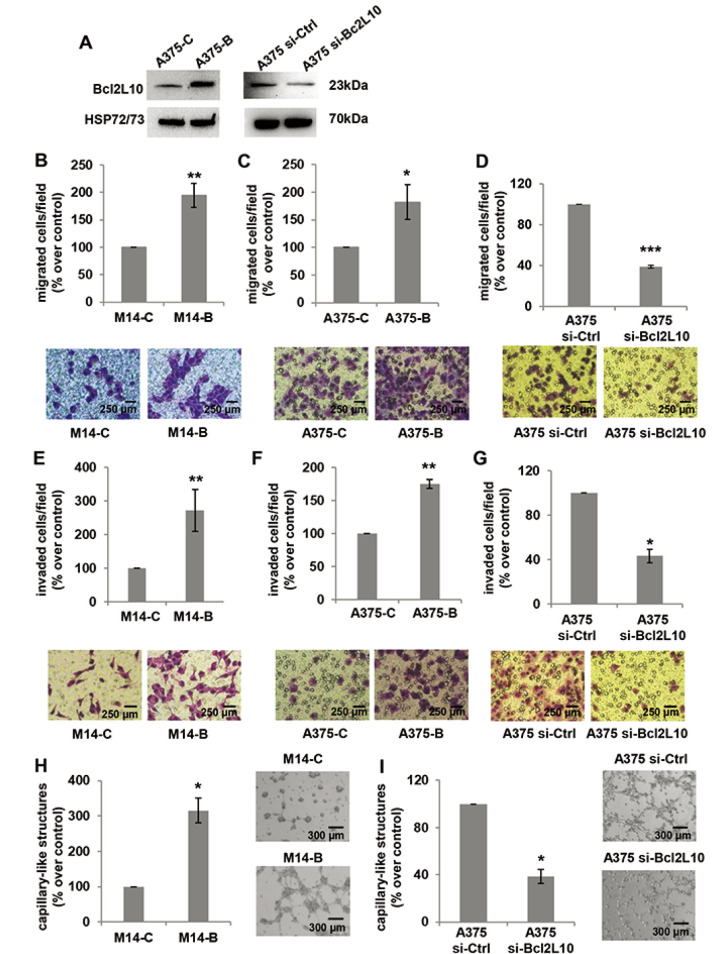
Bcl2L10 promotes *in vitro* melanoma cell invasion, migration and VM. (A) Western blot analysis of Bcl2L10 protein expression in Bcl2L10 overexpressing (A375-B) and control (A375-C) A375 cells, and A375 cells transfected with si-Bcl2L10 (A375 si-Bcl2L10) or si-Ctrl (A375 si-Ctrl). Representative images of three independent experiments with similar results. HSP72/73 was employed to check equal loading and transfer. (B–D) Bar charts and representative images of *in vitro* cell migration of (B) M14 cells stably overexpressing Bcl2L10 protein (M14-B) and relative control (M14-C), (C) A375-C and A375-B cells, and (D) A375 si-Bcl2L10 or A375 si-Ctrl. (E–G) Bar charts and representative images of *in vitro* cell invasion of (E) M14-C and M14-B cells, (F) A375-C and A375-B cells, and (G) A375 si-Bcl2L10 or A375 si-Ctrl. Each condition was analyzed in technical duplicate in two (D, F, G) or three (B, C, E) independent experiments. (B–G) Data are reported as the percentage of migrated or invaded cells respected to control. (H, I) Quantification and representative images of capillary-like structure formation in (H) M14-B and M14-C cells and (I) in A375 si-Bcl2L10 and A375 si-Ctrl cells. Each condition was analyzed in technical duplicate in three (H) or two (I) independent experiments. Values are expressed as percentage of capillary-like structures formed in M14-B or A375 si-Bcl2L10 cells *versus* relative controls. (B–I) Data were expressed as mean ± standard deviation. Scale bars have been reported. Statistical analysis was performed applying unpaired two-tailed student’s *t* test with Welch’s correction. * *P* < 0.05; ** *P* < 0.01; *** *P* < 0.001

Next, we investigated the role of Bcl2L10 in *in vitro* proliferation, clonogenic ability and *in vivo* tumor growth. Although previously published data demonstrated Bcl2L10 ability to affect proliferation of ovarian [18], gastric [19] and hepatocellular [13] carcinoma, we did not observe any effect of Bcl2L10 either on proliferation or clonogenic ability of melanoma cells (Figure S1). Different from the findings evidencing Bcl2L10 as a tumor growth inhibitor in hepatocellular carcinoma [13], and in agreement with our *in vitro* results obtained both in this paper and in the one previously published [11], *in vivo* experiments demonstrated that Bcl2L10 does not affect *in vivo* tumor growth of M14 xenografts (Figure S2). We next evaluated the involvement of Bcl2L10 in several steps during tumor progression, such as cell migration, cell invasion, and VM (Figure 2). To achieve this purpose, we performed *in vitro* transwell migration and invasion assays, finding that *Bcl2L10* overexpression induced a significant increase of both cell migratory and invasive capacity of M14 cells (Figure 2B, 2E). The ability of Bcl2L10 to affect *in vitro* cell migration and invasion was confirmed in A375 cells transiently transfected with *Bcl2L10* overexpressing plasmid (A375-B cells) (Figure 2A, 2C, 2F) or with specific small interference RNA smart pool targeting Bcl2L10 (si-Bcl2L10) (Figure 2A, 2D, 2G). Considering that in melanoma, the trans-differentiation of plastic tumor cells into a vasculogenic phenotype provides a tumor with a higher level of autonomy and, therefore, higher aggressiveness, we next explored Bcl2L10’s role in VM, and the process in which the vessels formed by tumor cells mimic endothelial cell functions [37]. When compared to control cells, *Bcl2L10* overexpressing cells showed enhanced VM, evaluated as percentage of capillary-like structures formed after seeding melanoma cells onto the gelled Cultrex BME (Figure 2H). In accordance with this result, we observed a significant reduction of percentage of capillary-like structures in Bcl2L10-silenced A375 cells respected to control ones (Figure 2I).

Bcl2L10 was found to inhibit angiogenesis of hepatocellular carcinoma *in vitro* and *in vivo* [13]. Thus, we investigated whether Bcl2L10 was able to affect *in vitro* and/or *in vivo* angiogenesis of melanoma models. Human umbilical vein cell line (EA.hy926) seeded on BME and exposed to CM derived from M14 cells overexpressing *Bcl2L10* formed a similar number of tubular-like structures when compared to EA.hy926 cells exposed to CM from M14 control cells (Figure S3). In agreement with the *in vitro* results, matrigel plugs containing CM from *Bcl2L10* overexpressing M14 cells injected in C57Bl/6 mice, showed similar haemoglobin content when compared to the matrigel plugs containing CM from control cells (Figure S3). In support of these results, analysis of vascular endothelial growth factor secretion by enzyme-linked immunosorbent assay indicated a non-significant induction (1.2 ± 0.14-fold) of vascular endothelial growth factor in Bcl2L10 transfectants, respect to control cells (*P* = 0.18). These results indicate that Bcl2L10 contributes to tumor microcirculation by increasing VM but not angiogenesis.

### *Bcl2L10* expression affects pathways involved in cell migration, invasion, and VM

**Figure 3.**
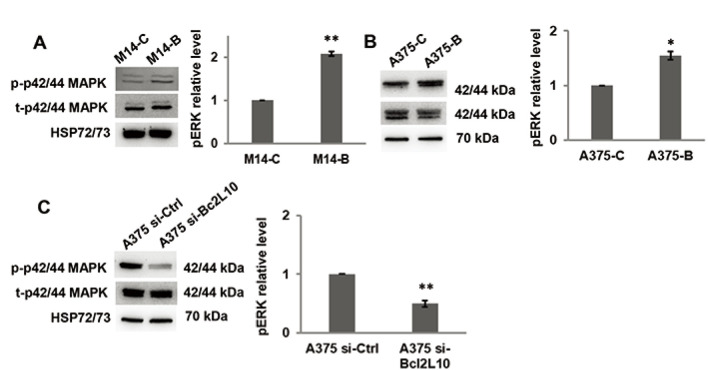
Bcl2L10 promotes ERK phosphorylation. (A–C) Western blot analysis of total (t) and phosphorylated (p) p44/42 MAPK (ERK1/2) and relative densitometric analysis of pERK in (A) M14-B and M14-C cells, (B) A375-C and A375-B cells, and (C) A375 si-Bcl2L10 and A375 si-Ctrl cells. Reported images are representative of three independent experiments with similar results. HSP72/73 was employed to check equal loading and transfer. For densitometric analysis, the relative levels of proteins were expressed in the histograms as fold changes respected to relative control after normalization. Data were expressed as mean ± standard deviation. Statistical analysis was performed applying unpaired two-tailed student’s *t* test with Welch’s correction. * *P* < 0.05; ** *P* < 0.01

**Figure 4.**
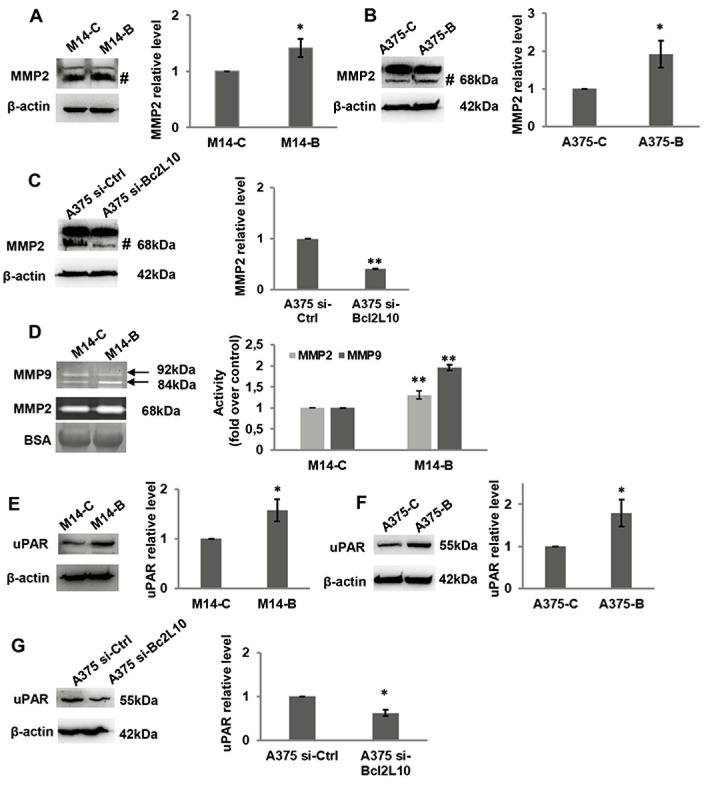
Bcl2L10 promotes metalloproteinases activities and increases uPAR level. (A–C) Western blot analysis and relative densitometric analysis of MMP2 protein expression in protein extracts of (A) M14 cells stably overexpressing *Bcl2L10* (M14-B) and relative control (M14-C), and in (B) A375 cells transfected with empty (A375-C) or *Bcl2L10* (A375-B) expressing vectors, or (C) with pooled oligonucleotide mix against Bcl2L10 (A375 si-Bcl2L10) or scramble target (A375 si-Ctrl). Active form of MMP2, 68 kDa, is indicated (^#^). (D) Gelatinase activity and relative densitometric analysis of metalloproteinases MMP2 and MMP9 enzymatic activity of CM from M14-B and M14-C cells incubated in serum free medium for 24 h. Active form of MMP2, 68 kDa, and pro- and active form of MMP9, 92 kDa and 84 kDa respectively, are indicated. CM were also analyzed by SDS-polyacrylamide gel electrophoresis, which is transferred to nitrocellulose membrane and bovine serum albumin signal (BSA) was visualized using ponceau staining. (A–D) Representative images of three independent experiments with similar results are shown. (A–C) β-actin primary antibody was used as loading and transferring control. For densitometric analysis, the relative levels of proteins were expressed in the histograms as fold changes respected to relative control after normalization. Data were expressed as mean ± standard deviation. Statistical analysis was performed applying unpaired two-tailed student’s *t* test with Welch’s correction. * *P* < 0.05; ** *P* < 0.01. BSA: bovine serum albumin signal

As several intracellular pathways, such as the ERK1/2 pathway, are known to be actively involved in cell migration and invasion, we examined the effect of Bcl2L10 modulation on phosphorylation of ERK1/2 (p42/44 MAPK). Overexpressing *Bcl2L10* in M14 (Figure 3A) and A375 (Figure 3B) cells increased levels of p42/44 ERK1/2 phosphorylation, while after Bcl2L10 silencing in A375 cells, a significant reduction of phosphorylated p42/44 ERK1/2 was observed (Figure 3C), indicating that this pathway is affected by Bcl2L10 modulation. Next, we evaluated whether Bcl2L10 was able to affect the activity of matrix metalloproteinases MMP2 and MMP9, which we previously demonstrated to be modulated by Bcl-2 and Bcl-xL in melanoma [4, 31]. These two zinc dependent proteases play a major role in proteolytic degradation of extracellular matrix components and promote tumor invasion and metastasis [38]. *Bcl2L10* overexpressing M14 and A375 cells increased the level of activated MMP2 protein respected to controls (Figure 4A, 4B). The Bcl2L10-dependent cleavage of MMP2 protein was confirmed in A375 cells after Bcl2L10 silencing (Figure 4C). In accordance with these results, increased enzymatic activity of MMP2, evaluated by gelatin zymography, was observed in CM from Bcl2L10 overexpressing M14 cells when compared to CM from control ones (Figure 4D). This experiment also allowed appreciating the concomitant activation of the secreted MMP9 in CM from Bcl2L10 overexpressing M14 cells respected to control ones.

Further, we evaluated whether *Bcl2L10* was able to affect the expression of uPAR, a key regulatory molecule of migration, invasion, and VM [39]. As shown by western blot analysis, we observed an increased level of uPAR protein in Bcl2L10 overexpressing M14 (Figure 4E) and A375 (Figure 4F) cells, while a reduced level of uPAR was detected upon Bcl2L10 silencing in A375 cells (Figure 4G).

**Figure 5.**
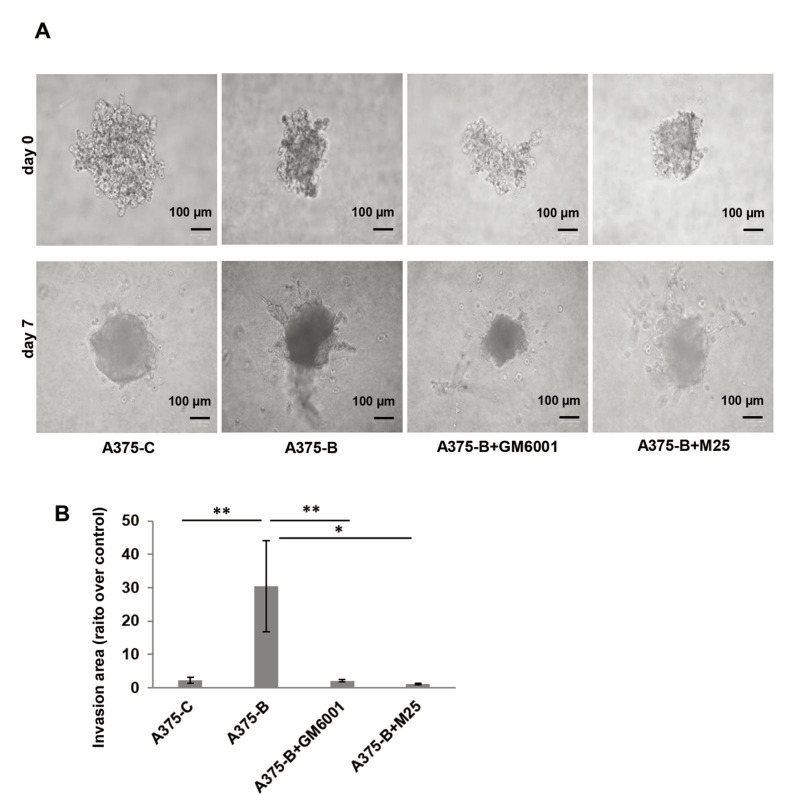
Bcl2L10 enhances the ability of melanoma cells to invade. (A) Representative images of 3D spheroid invasion assays using A375 cells transfected with empty (A375-C) or *Bcl2L10* (A375-B) expressing vectors over 7 days following the addition of invasion matrix, and A375-B cells treated with matrix metalloproteinases (MMPs) inhibitor (Ilomastat/GM6001) or uPAR inhibitor (peptide M25). (B) Histograms, quantification of 3D invasion relative to invasion area. Data were expressed as mean ± standard error of the mean. Statistical analysis was performed applying one-way analysis of variance, Tukey post-hoc analysis. * *P* < 0.05; ** *P* < 0.01

We also assessed the invasive capability in a 3D cell culture system using collagen matrix-embedded tumor spheroids derived from A375 cells (Figure 5). *Bcl2L10* transiently overexpressing A375 cells (A375-B) enhanced invasion into the surrounding matrix and radial out-growth compared to control cells (A375-C), which is impaired by both Ilomastat/GM6001, a synthetic pan-inhibitor of matrix metalloproteinases, and M25, a synthetic peptide impairing uPAR functions [33, 35, 40]. M25 application has already validated both by other and us to study the biological role of uPAR *in vitro* 2D cell migration/invasion, VM, and 3D invasion assay [33–35].

Our data obtained in 3D model demonstrate that both MMPs and uPAR mediate the effect of Bcl2L10 in cellular invasion.

**Figure 6.**
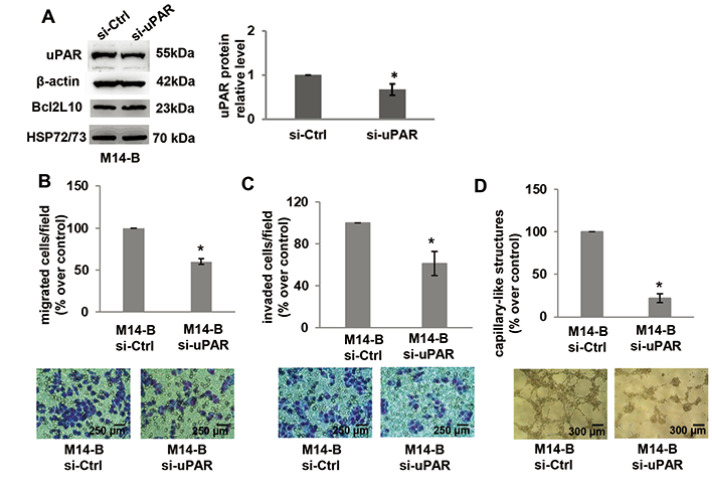
Bcl2L10 promotes migration, invasion, and VM of melanoma cells through uPAR. (A) Western blot analysis of uPAR and Bcl2L10 and densitometric analysis of uPAR protein expression in Bcl2L10 overexpressing M14 clone (M14-B) transfected with siRNA oligonucleotides against uPAR (si-uPAR) or scramble (si-Ctrl) target sequences. Reported images are representative of three independent experiments with similar results. β-actin and HSP72/73 were employed to check equal loading and transfer. For densitometric analysis, protein levels were quantified by using ImageJ software and the relative levels of proteins were expressed in the histograms as ratio changes respect to relative control after normalization. Data were expressed as mean ± standard deviation. Statistical analysis was performed applying corrected *t* test. (B–D) Quantification and representative images of *in vitro* cell (B) migration, (C) invasion, and (D) capillary-like structure formation in M14-B cells transfected with siRNA oligonucleotides against uPAR (si-uPAR) or scramble (si-Ctrl) target sequences. Values are expressed as a percentage of migrated/invaded cells or capillary-like structure formed respect to control. (B–D) Each condition was analyzed in duplicate in three (C) or two (B, D) independent experiments. Data were expressed as mean ± standard deviation. Scale bars have been reported. Statistical analysis was performed applying unpaired two-tailed student’s *t* test with Welch’s correction. * *P* < 0.05

To more deeply characterize the relevance of uPAR in the ability of Bcl2L10 to affect *in vitro* properties associated with melanoma aggressiveness, cell migration/invasion and VM were analyzed after uPAR downregulation in M14 cells stably overexpressing *Bcl2L10* by using a specific small interference RNA smart pool (si-uPAR), which is able to reduce both uPAR mRNA and protein expression. As expected, uPAR silencing did not affect both Bcl2L10 protein and mRNA expression (Figure 6A, Figure S4). Downregulation of uPAR levels strongly reduced *in vitro* cell migration/invasion and VM of *Bcl2L10* overexpressing melanoma cells (Figure 6B–D). In order to confirm these results, we performed *in vitro* cell migration, cell invasion, and VM assays after pharmacological inhibition of uPAR with M25 peptide. As shown in Figure S5, M25 peptide significantly reduced cell migration, invasion, and capillary-like structure formation. These results demonstrate that uPAR plays a role in Bcl2L10-dependent promotion of cell migration/invasion, and VM.

## Discussion

Here we demonstrated the ability of Bcl2L10 to promote a more aggressive *in vitro* phenotype in melanoma models. In particular, in accordance with data demonstrating *Bcl2L10* expression in human melanoma cells and melanoma patient specimens [11], we confirmed the expression of endogenous Bcl2L10 protein in a panel of melanoma cells. Of note, by using human melanoma cells stably overexpressing *Bcl2L10* or transiently transfected for loss/gain function of Bcl2L10, we evidenced that Bcl2L10 is an inducer of *in vitro* cell invasion/migration and VM, similarly to what was observed for other anti-apoptotic proteins, such as Bcl-2 and Bcl-xL [4, 31]. The ability of Bcl2L10 to promote *in vitro* cell invasion was demonstrated by using both 2D assays and 3D culture of tumor spheroids, the latter recapitulating *in vivo* human solid tumors.

By western blotting and zymography assays, we showed that MMP2 and MMP9 protein activities were increased by Bcl2L10. MMP family plays a relevant role in the degradation of extracellular matrix components and basement membrane [38]. Among the MMPs, MMP2 and MMP9 represent the most important proteins in metastasis [41]. The use of a MMPs inhibitor in 3D spheroid invasion assay reduced invasiveness of melanoma cells overexpressing Bcl2L10 protein, pointing out the role of MMPs in mediating Bcl2L10 function.

In addition to MMPs, uPAR also acts as a proteolytic degrader of extracellular matrix [42]. Interestingly, we observed a remarkable reduction of radial out-growth of *Bcl2L10* overexpressing cells in 3D culture system after pharmacological inhibition of uPAR. In addition, a significant reduction of both cell migration and invasion in 2D assays after both uPAR mRNA downregulation using RNA interference and pharmacological blocking has been observed. These data indicate that, in addition to MMPs, uPAR also could play a role in promoting Bcl2L10-dependent migration and invasion functions. Several studies support the relevance of the plasminogen activator system in melanoma: urokinase plasminogen expression correlates with the metastatic potential of melanoma models, and the expression of urokinase plasminogen and its cognate receptor uPAR are increased in late-stage melanocytic tumors [43]. Other studies support a direct involvement of uPAR in the melanoma progression: hypoxia promotes lymph-node metastasis in human melanoma xenografts by upregulating uPAR [44]. Moreover, inhibition of uPAR through RNA interfering reduces tumor growth and induces pro-apoptotic effects in melanoma models with acquired resistance to target therapy [45, 46].

In this study we also observed that the modulation of Bcl2L10 also induces phosphorylation of ERK1/2, a relevant pathway in melanoma pathobiology. In fact, high ERK1/2 phosphorylation levels have been detected in clinical melanoma metastases and various melanoma cell lines [47], and combination of BRAF and mitogen-activated protein kinase inhibition is the elective treatment in patients with advanced BRAF-mutant melanoma.

Moreover, we evidenced the ability of Bcl2L10 to promote the formation of VM, which is a phenomenon indicating the *de novo* formation of vasculogenic-like networks by aggressive tumor cells, and correlating with high tumor grade, short survival, invasion, and metastasis [48, 49]. Of note, in this work, the formation of capillary-like structures was impaired by both genetic and pharmacological targeting of uPAR, thus demonstrating the role played by uPAR in Bcl2L10-dependent VM. Several molecular mechanisms and signal pathways are responsible of VM induction and formation [48, 50]. Among the others, increased activation of ERK1/2 and MMP2, as well as upregulation of MMP14, have been reported in melanoma cells [51]. Moreover, positive correlation of uPAR expression with VM formation, metastasis, and poor prognosis in different aggressive cancers has been reported [49]. In this content, we previously evidenced that inhibition of uPAR expression, by genetic approach in melanoma cells, decreased the *in vitro* VM formation induced by miR-378a-5p. More recently, it has been reported that uPAR targeting by M25 peptide could impair capillary-like formation in drug-resistant melanoma cells [34]. Moreover, we demonstrated that Bcl-xL was also able to induce VM in both *in vitro* and *in vivo* model of melanoma [4].

Although we demonstrated a relevant involvement of Bcl2L10 in promoting migration, invasion and formation of capillary-like structures, we did not observe any substantial effect of Bcl2L10 in the regulation of *in vitro* clonogenic ability and, in accordance with our previously published data [11], of cell proliferation. Unlike our results, Bcl2L10 knockdown has been reported to promote the proliferation of ovarian [18] and gastric [19] cancer cells. Moreover, ectopic expression of *Bcl2L10* in hepatocellular carcinoma models suppressed both cell viability and colony formation [13]. In agreement with our *in vitro* results, we found that *in vivo* tumor growth was not affected by Bcl2L10. On the contrary, overexpression of *Bcl2L10* inhibited the growth of hepatocellular carcinoma xenografts and reduced experimental lung metastasis [13]. In addition, forced expression of *Bcl2L10* in hepatocellular carcinoma model reduced *in vitro* and *in vivo* angiogenesis [13], while we did not observe significant differences *in vitro* and *in vivo* angiogenesis assay after *Bcl2L10* forced expression. On the basis of these evidence, we can suggest that the effect of Bcl2L10 is strictly depended on the tumor histotype.

We have previously demonstrated that Bcl2L10 is an anti-apoptotic protein in melanoma, where it is able to protect cells from the cytotoxic effect of different drugs, such as cisplatin, dacarbazine, ABT-737 (a pan Bcl-2 inhibitor) and PLX-4032 (a BRAF inhibitor, administered alone or in combination) [11]. In accordance with our previous results demonstrating the anti-apoptotic activity of Bcl2L10 [11], here we found a decreased [Ca^2+^]_i_ in melanoma cells overexpressing *Bcl2L10*. These results agree with those obtained in breast cancer, where Bcl2L10 has been reported to promote its anti-apoptotic function by negatively regulating the release of calcium from the endoplasmic reticulum through the interaction of the Bcl-2 homology (BH)4 domain with inositol-1,4,5-trisphosphate receptor [12]. In addition to proliferation and cell death, intracellular calcium dynamics regulate many cellular processes including cytoskeleton remodeling and cell migration [52].

Bcl-2 proteins have been reported to be localized at the mitochondria-endoplasmic reticulum interface and to interact with key channels or receptors on both endoplasmic reticulum and mitochondrial membranes. Thus, the role of Bcl-2 proteins in regulating intracellular and mitochondrial Ca^2+^ homeostasis, and subsequently to several other processes such as cell migration, independently of apoptosis, is emerging [53]. Increased intracellular Ca^2+^ levels have been described to be associated to the anti-migration effects of alkaloid compounds in liver cancer cells [54]. Based on this consideration, we can hypothesize that the increased migration properties observed in melanoma cells overexpressing *Bcl2L10* could also be linked to the reduced [Ca^2+^]_i_ detected in the same cells.

Due to their multiple functions in cancer, Bcl-2 family proteins have become interesting targets for anti-cancer drugs [55]. The recent approval for clinical practice of venetoclax, a BH3 mimetic specific for Bcl-2, corroborated the clinical relevance of using anti-apoptotic proteins as therapeutic targets, not only for hematologic malignancies but also for solid tumors. In fact, numerous clinical trials are ongoing to evaluate the effects of specific anti-apoptotic Bcl-2 proteins or pan inhibitors as single agents or in combination therapy in solid tumors [55]. Different factors contribute to cellular responsiveness to BH3 mimetic drugs. Among these factors, the differential “addiction” to anti-apoptotic (mainly Bcl-2, Bcl-xL or Mcl-1) proteins has been showed. The concept of “addiction” has been introduced to explain different survival of tumor cells depending on the expression levels of these proteins [56, 57]. Taking into account of our results evidencing not only the anti-apoptotic function of Bcl2L10 but also its role in promoting *in vitro* melanoma cell migration, invasion, and VM through the involvement of MMPs and uPAR, we can suggest that Bcl2L10 should be considered in the evaluation of novel anti-apoptotic Bcl-2 protein inhibitors and could represent a potential target for the treatment of melanoma.

## Abbreviations

[Ca^2+^]_i_: intracellular calcium levels
2D: two-dimensional
3D: three-dimensional
Bcl-2: B-cell lymphoma-2
Bcl2L10: B-cell lymphoma-2-like protein-10
BH: B-cell lymphoma-2 homology domain
BME: basement membrane extract
BRAF: v-raf murine sarcoma viral oncogene homolog B1
CM: cultured medium
ERK: extracellular-signal-regulated kinase
FURA-2-AM: 2-[6-[bis[2-[(Acetyloxy)methoxy]-2-oxoethyl]amino]-5-[2-[2-[bis[2-[(acetyloxy)methoxy]-2-oxoethyl]amino]-5-methylphenoxy]ethoxy]-2-benzofuranyl]-5-oxazolecarboxylic acid (acetyloxy)methyl ester
HSP: heat shock protein
MAPK: mitogen-activated protein kinases
MMPs: matrix metalloproteinases
SDS: sodium dodecyl sulphate
SFM: serum free medium
uPAR: urokinase plasminogen activator receptor
VM: vasculogenic mimicry

## Supplementary materials

The supplementary material for this article is available at: https://www.explorationpub.com/uploads/Article/file/100268_sup_1.pdf.

## Declarations

### Copyright

© The Author(s) 2022.
