# Supplementary material for: Bcl-2-like protein-10 increases aggressive features of melanoma cells
Source: Explor Target Antitumor Ther. 2022 Jan 30;3(1):11–26. doi: 10.37349/etat.2022.00068 (PMC9400776; doi:10.37349/etat.2022.00068)
Supplement: Supplementary file 1 [file etat-03-100268-s001.pdf]

Figure S1

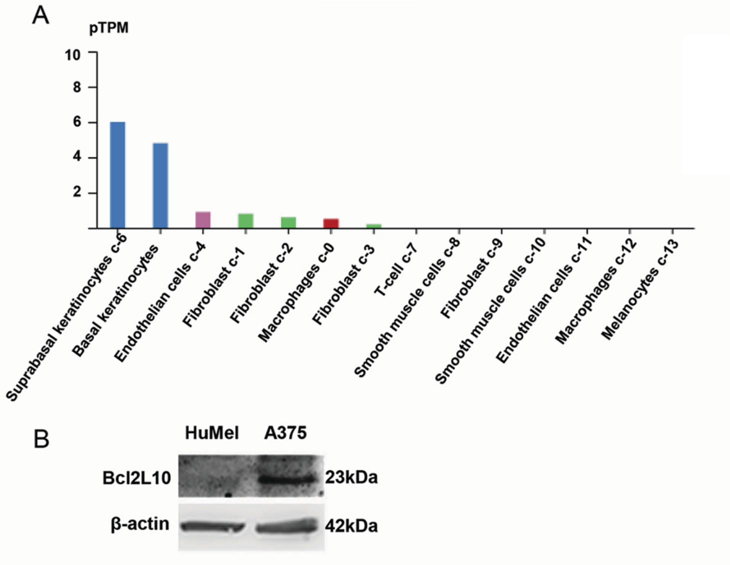

Figure S1. Expression of Bcl2L10 in melanocytes. A) Bar charts showing the expression of Bcl2L10 mRNA in normal cell types. Data are from The Human Protein Atlas database (https://www.proteinatlas.org/). B) Western blot analysis of endogenous Bcl2L10 protein expression in immortalized human melanocyte cell line (HuMel) and human melanoma cell line (A375). HuMel cells were kindly provided by Dr. David Fisher (Massachusetts General Hospital, Boston). Invitrogen PA5-22190 antibody against Bcl2L10 was used. β-actin was used to check equal loading and transfer.

Figure S2

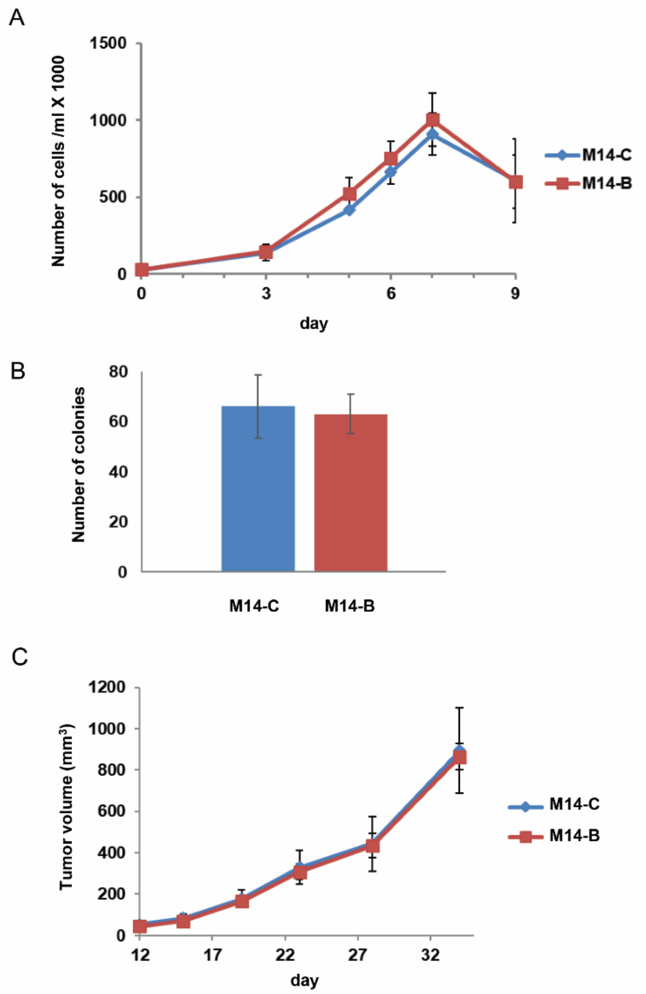

Figure S2. **Bcl2L10 expression in M14 cells does not affect neither *in vitro/in vivo* tumor growth nor colony formation ability**. Evaluation of (**A**) *in vitro* cell proliferation, (**B**) colony formation ability, and (**C**) *in vivo* tumor growth of M14 cells stably overexpressing Bcl2L10 (M14-B) or M14 control cells (M14-C). (**A**) Cells (3 x 10^4) were plated in 60-mm Petri. At fixed end points cells were trypsinized, mixed with trypan blue and the number of viable cells was counted using a haemocytometer; (**B**) To evaluate the cell colony-forming ability, 5 × 10^2 M14-C and M14-B cells were seeded into 60-mm Petri dishes for 10 days. Colonies were stained with 2% methylene blue in 95% ethanol and counted (1 colony>50 cells); (**C**) Cells in exponential growth phase were harvested from the culture, washed, and resuspended in PBS and injected subcutaneously into nude mice at 5 × 10^6 viable cells/mice. Tumor weight was monitored and calculated as previously reported [1]. Female CD-1 nude (nu/nu) mice, 6–8 weeks old and 22–24 g in body weight were purchased from Charles River Laboratories (Calco, Italy). All procedures involving animals and their care were authorized and certified by D.lgs 26/2014 (816/2015- PR del 11/08/2015) of the Italian Minister of Health.

(1) Trisciuoglio D, Desideri M, Ciuffreda L, Mottolese M, Ribatti D, Vacca A, et al. Bcl-2 overexpression in melanoma cells increases tumor progression-associated properties and in vivo tumor growth. J Cell Physiol 2005;205:414-21.

Figure S3

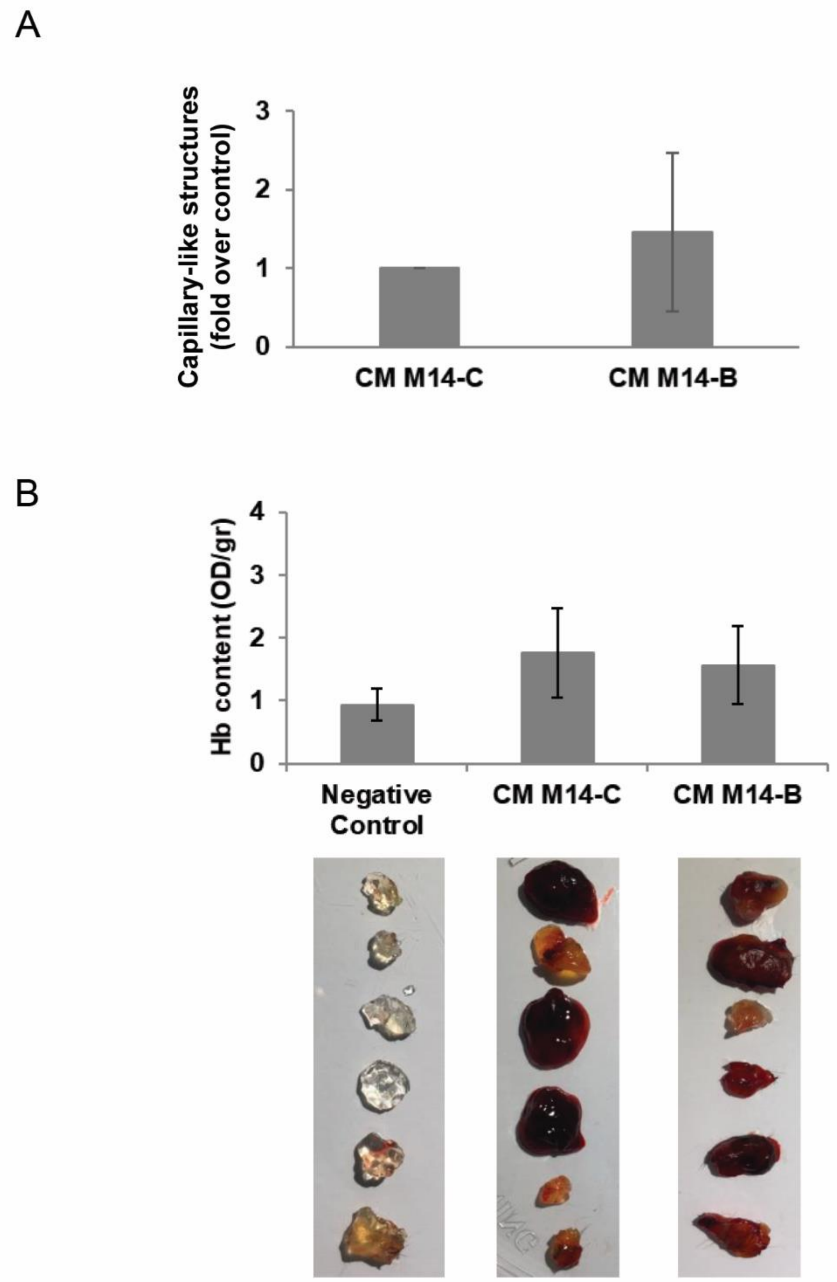

Figure S3. Bcl2L10 does not affect *in vitro* or *in vivo* angiogenesis. (**A**) Endothelial capillary tube-like network formation (morphogenesis) evaluated in EA.hy926 cells seeded on Cultrex BME and exposed to cultured medium (CM) derived from M14 cells stably overexpressing Bcl2L10 (M14-B) or M14 control cells (M14-C). The average ± SD of four independent experiments performed in duplicate is reported. Statistical analysis was performed applying t-test. 250ul of polymerized Cultrex BME (12–18 mg/ml) were added to each well of precooled 24-well tissue culture plate. The plate was incubated at 37 °C for 1h to allow the matrix solution to solidify. A total of 2 × 10^5 EA.hy926 cells, were seeded on BME and exposed to CM derived from M14-C and M14-B cells. After 6h, capillary tube-like network formation was observed as reported for melanoma cells (**Figure 2 and 6**). (**B**) Bar chart (upper panel) and representative images (lower panel) showing

quantification results of the haemoglobin content in the Matrigel plugs containing CM from M14-C or M14-B cells. In the negative controls the CM was replaced with serum free medium. *In vivo* Matrigel assay and quantification of the haemoglobin content in the Matrigel plugs were done as previously reported [2] using CM from 5×10^6 viable M14-C or M14-B cells. Groups of 6 female mice were used for each experimental point. All procedures involving animals and their care were conducted as reported in Supplementary Figure S1.

References

(2) Gabellini C, De Luca T, Trisciuoglio D, Desideri M, Di Martile M, Passeri D, et al. BH4 domain of bcl-2 protein is required for its proangiogenic function under hypoxic condition. Carcinogenesis 2013;34:2558-67

Figure S4

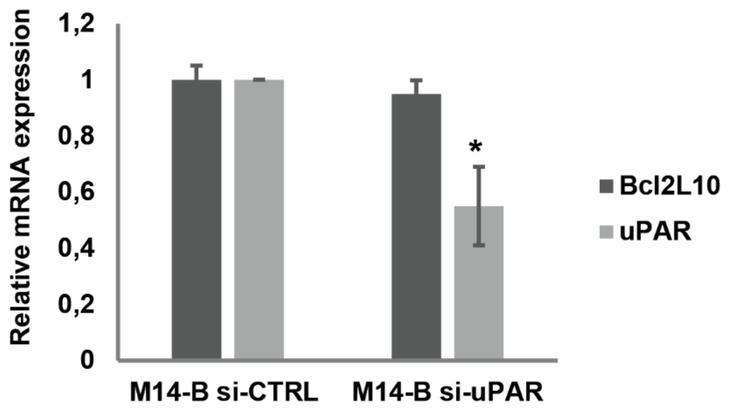

Figure S4. Rt-PCR analysis of uPAR and Bcl2L10 mRNA. mRNA levels determined by RT-PCR in M14-B cells transfected with siRNA scramble (M14-B si-CTRL) or siRNA uPAR (M14-B si-uPAR). Values are expressed as means of ratio±SD, where 'ratio' was calculated considering siRNA uPAR versus siRNA scramble cells. Total RNA was extracted using a Qiagen RNeasy Mini kit (Qiagen) according to the manufacturer's instructions. Reverse transcription was performed using RevertAid Reverse Transcriptase (Thermo Scientific). RT-PCR was performed using the SYBR green dye detection method and or beta-actin mRNA for normalization. Primers used were: beta-actin For 5'- ATTGCCGACAGGATGCAGAA-3'; beta-actin Rev 5'-GCTGATCCACATCTGCTGGAA-3'; Bcl2L10 For 5'-GCCTTCATTTATCTCTGGACAC-3'; Bcl2L10 Rev 5'-AAGGTGCTTTCCCTCAGTTC-3'; uPAR For 5'-ATCACCAGCCTTACCGAGGTT-3'; uPAR Rev 5'-ATGCATTCGAGGTAACGGC-3'. Values are reported as ratio respect to control cells using the 2–ΔΔCt method. *p < 0.05;

Figure S5

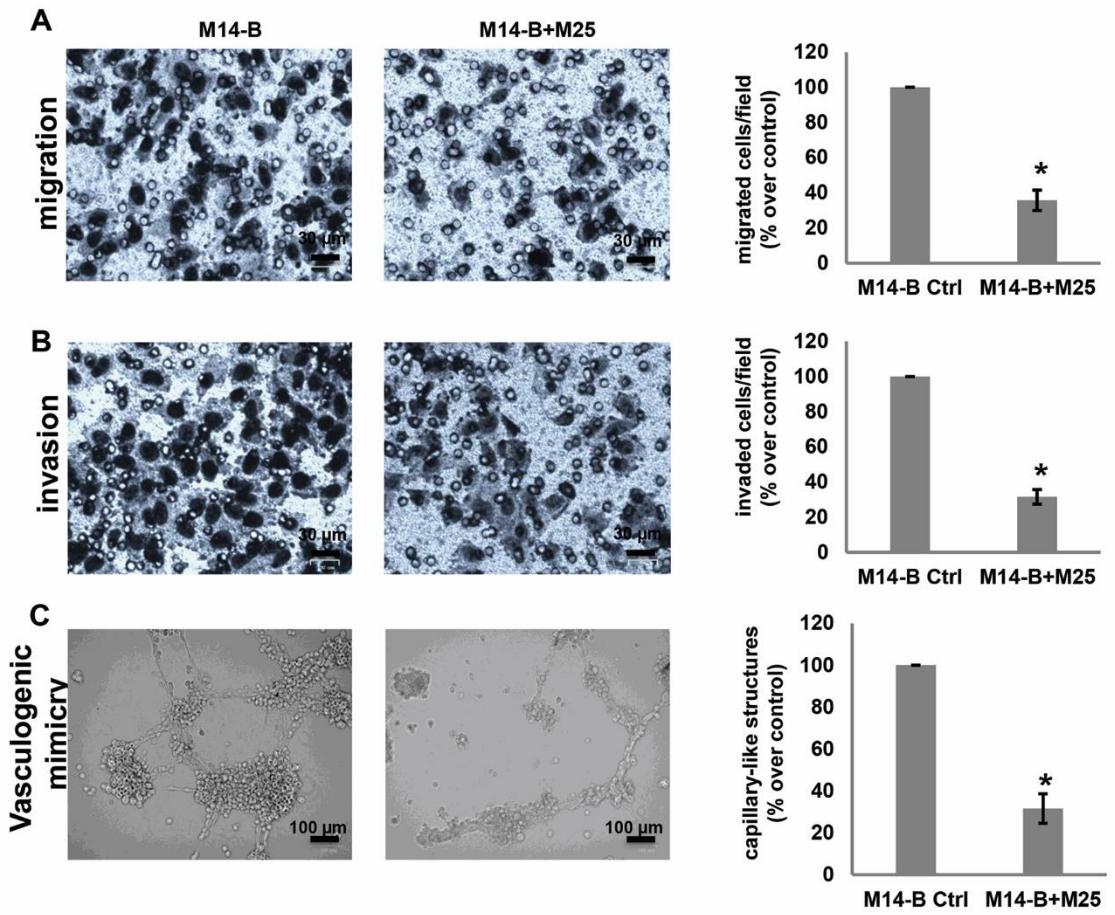

Figure S5. Bcl2L10 promotes migration, invasion and vasculogenic mimicry of melanoma cells through uPAR (A-C) Representative images and quantification of in vitro cell (A) migration, (B) invasion and (C) capillary-like structure formation in M14-B cells after pharmacological inhibition of uPAR by using M25 peptide. Values are expressed as a percentage of migrated/invaded cells or capillary-like structure formed respect to control. Data were expressed as mean ± standard deviation. Scale bars have been reported. Statistical analysis was performed applying unpaired two-tailed Student's t-test with Welch's correction. *p-values< 0.05.
